# Transcriptomic Analysis Reveals That Granulocyte Colony-Stimulating Factor Trigger a Novel Signaling Pathway (TAF9-P53-TRIAP1-CASP3) to Protect Retinal Ganglion Cells after Ischemic Optic Neuropathy

**DOI:** 10.3390/ijms23158359

**Published:** 2022-07-28

**Authors:** Rong-Kung Tsai, Keh-Liang Lin, Chin-Te Huang, Yao-Tseng Wen

**Affiliations:** 1 Institute of Eye Research, Hualien Tzu Chi Hospital, Buddhist Tzu Chi Medical Foundation, Hualien 970473, Taiwan; tsai.rk@gmail.com (R.-K.T.); chintehuang@hotmail.com (C.-T.H.); 2 Institute of Medical Sciences, Tzu Chi University, Hualien 970374, Taiwan; 3 Doctoral Degree Program in Translational Medicine, Tzu Chi University and Academia Sinica, Hualien 970374, Taiwan; 4 Department of Optometry, Mackay Medical College, New Taipei City 252005, Taiwan; eye2020@mmc.edu.tw; 5 Department of Ophthalmology, Chung Shan Medical University Hospital, College of Medicine, Chung Shan Medical University, Taichung 402306, Taiwan

**Keywords:** rat anterior ischemic optic neuropathy model, retinal ganglion cell death, TBP associated factor 9, TP53 regulated inhibitor of apoptosis 1, transcriptome

## Abstract

Optic nerve head (ONH) infarct can result in progressive retinal ganglion cell (RGC) death. The granulocyte colony-stimulating factor (GCSF) protects the RGC after ON infarct. However, protective mechanisms of the GCSF after ONH infarct are complex and remain unclear. To investigate the complex mechanisms involved, the transcriptome profiles of the GCSF-treated retinas were examined using microarray technology. The retinal mRNA samples on days 3 and 7 post rat anterior ischemic optic neuropathy (rAION) were analyzed by microarray and bioinformatics analyses. GCSF treatment influenced 3101 genes and 3332 genes on days 3 and 7 post rAION, respectively. ONH infarct led to changes in 702 and 179 genes on days 3 and 7 post rAION, respectively. After cluster analysis, the levels of TATA box-binding protein (TBP)-associated factor were significantly reduced after ONH infarct, but these significantly increased after GCSF treatment. The network analysis revealed that TBP associated factor 9 (TAF9) can bind to P53 to induce TP53-regulated inhibitor of apoptosis 1 (TRIAP1) expression. To evaluate the function of TAF9 in RGC apoptosis, GCSF plus TAF9 siRNA-treated rats were evaluated using retrograde labeling with FluoroGold assay, TUNEL assay, and Western blotting in an rAION model. The RGC densities in the GCSF plus TAF9 siRNA-treated rAION group were 1.95-fold (central retina) and 1.75-fold (midperipheral retina) lower than that in the GCSF-treated rAION group (*p* < 0.05). The number of apoptotic RGC in the GCSF plus TAF9 siRNA-treated group was threefold higher than that in the GCSF-treated group (*p* < 0.05). Treatment with TAF9 siRNA significantly reduced GCSF-induced TP53 and TRIAP1 expression by 2.4-fold and 4.7-fold, respectively, in the rAION model. Overexpression of TAF9 significantly reduced apoptotic RGC and CASP3 levels, and induced TP53 and TRIAP1 expression in the rAION model. Therefore, we have demonstrated that GCSF modulated a new pathway, TAF9-P53-TRIAP1-CASP3, to control RGC death and survival after ON infarct.

## 1. Introduction

In elderly individuals, the most common type of acute optic neuropathy is non-arteritic anterior ischemic optic neuropathy (NAION), with an estimated annual incidence of 3.72 per 100,000 individuals in Taiwan [1]. NAION is defined clinically as painless visual loss with swelling of the optic disc leading to optic disc atrophy [2]. Currently, there is no effective treatment for NAION. Optic nerve (ON) ischemia induces a series of detrimental events, eventually resulting in retinal ganglion cell (RGC) loss [2]. RGC death and axon degeneration are major complications of ischemic damage and mainly caused by oxidative stress [3,4,5,6], pro-inflammatory factors [6,7], aberrant calcium ion homeostasis [8], and macrophage polarization [7,9]. Therefore, a comprehensive investigation on the complex molecular mechanisms of axonal degeneration and RGC death in ON ischemia may bring about new possibilities for treatment.

Several efforts in preventing ON injuries and RGC death have been made using different approaches, such as anti-inflammatory compounds [10,11], neurotropic factors [12,13], oxidative stress regulators [5], calcium channel blockers [14], microglial activation inhibitors [7,15], and blood-borne macrophage infiltration blockers [7,12,16]. These potential treatments provide some possible directions to elucidate how to control the fate of RGCs. In context with our previous study on the neuroprotective effects of granulocyte colony-stimulating factor (GCSF), we found that GCSF exhibited the ability to rescue RGC from apoptosis, which may be involved in modulations of the blood–ON barrier, macrophage infiltration, and inflammatory reactions [7]. Moreover, our previous findings demonstrate that treatment with GCSF can activate the PI3K/AKT pathway to protect RGC from apoptosis [17].

GCSF is a member of the hematopoietic growth factor family. It is widely used in clinical practice for the treatment of neutropenia [18]. Recent findings have suggested that GCSF also has a nonhematopoietic role in memory improvement in Alzheimer’s disease and a neuroprotective role in Parkinson’s disease [19,20]. It also promotes angiogenesis and inhibits apoptosis and inflammation in rats with ischemic stroke [21,22,23]. Furthermore, the GCSF receptor (GCSFR) is expressed in various neural and glial cells, such as RGCs, microglia, astrocyte, and oligodendrocyte, which results in the direct activation of GCSFR signaling pathways, including JAK/STAT, PI3K/AKT, and MAPK/ERK pathways [24]. These signaling pathways are involved in cell growth and differentiation. Thus, the mechanisms by which GCSF promotes RGC survival are likely complex.

To deeply investigate and characterize the factors triggered by CGSF treatment, transcriptome analysis of the rat retina is a possible approach that can be used to investigate the transcriptome changes after ON infarct with or without GCSF treatment. The microarray technique is a method of high-throughput analysis to determine the expression levels of large numbers of genes belonging to specific pathways simultaneously. This technique has also been used to evaluate changes after axonal injury in both the retina and isolated RGCs [25,26]. Therefore, we considered that microarray analysis is an adequate tool to explore any possible mechanisms involved in RGC apoptosis and survival. 

Different animal models have been used to investigate RGC pathology. Herein, we used a rat anterior ischemic optic neuropathy (rAION) model as it represents similar features and pathology to human and primate AION [27]. The rAION model was achieved by photodynamic therapy, which generates superoxide radicals in the ON capillaries, causing capillary thrombosis, inflammation, and oxidative stress [27]. These pathological changes are important events to induce RGC apoptosis. Thus, this is an appropriate model to use in investigating the mechanisms of RGC apoptosis. 

In the present study, a comparative microarray analysis was adopted to explore the dynamic transcriptome changes in the rat retina under ON infarct and GCSF treatment. We identified that the mRNA levels of several TATA-box-binding protein (TBP)-associated factors (TAFs) were significantly reduced after ONH infarct, but significantly increased after GCSF treatment. Among these genes, we targeted one of the most upregulated, transcription initiation factor TFIID subunit 9 (taf9), which encodes for one of the smaller subunits of transcription factor IID (TFIID) that binds to the general transcription factor transcription initiation factor IIB (Gtf2b) and several transcriptional activators, such as p53 and Vp16 [28,29]. Taf9 physically interacts with p53 at its *N*-terminus, where p53 also interacts with its negative regulator, Mdm2, thereby inhibiting Mdm2 degradation of p53 [30]. Functionally, this interaction translates to an increase in p53-induced angiogenesis, DNA repair, cell arrest, cell survival, or apoptosis [30]. However, it is questionable whether TAF9 drives P53 toward cell survival or apoptosis. Thus, this study aimed to reveal the underlying mechanisms of TAF9 in RGC apoptosis and cell survival.

## 2. Results

### 2.1. Identification of Differentially Expressed Genes by Microarray

**Figure 1:**
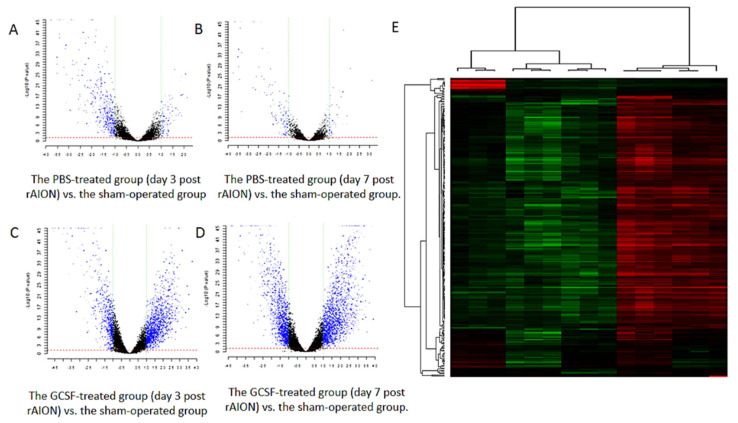
Gene expression profiles of the retina samples. (**A**–**D**) Volcano plot showing the differentially expressed genes in the PBS- and GCSF-treated groups. (**A**) The PBS-treated group (day 3 post rAION) vs. the sham-operated group. (**B**) The PBS-treated group (day 7 post rAION) vs. the sham-operated group. (**C**) The GCSF-treated group (day 3 post rAION) vs. the sham-operated group. (**D**) The GCSF-treated group (day 7 post rAION) vs. the sham-operated group. For each plot, the X-axis represents log2 FC, and the Y-axis represents −log10 (*p*-values). The differentially expressed genes are shown as red dots. (**E**) The heatmap of the hierarchical clustering of the differentially expressed genes. Up- and downregulated genes are represented in red and green colors, respectively. The differentially expressed genes were defined as having absolute FC > 1.5 and FDR < 0.1.

To investigate RGC death and survival at the transcriptional level, the rAION-inducted rats were treated with phosphate-buffered saline (PBS) or GCSF. The transcriptome profiles were analyzed using oligonucleotide microarrays. Microarray data were analyzed using the Gene Expression Pattern Analysis Suite to identify the differentially expressed genes. From a total of 24,358 analyzed genes, 3101 and 3332 transcripts were regulated by GCSF treatment on days 3 and 7 post rAION, respectively. In addition, 702 and 179 transcripts were regulated by PBS treatment on days 3 and 7 post rAION, respectively (Figure 1A–D). Unsupervised hierarchical clustering analysis of differentially expressed genes from all groups was conducted to investigate the similarity of the whole gene expression between the experimental samples. The result indicated that the profile of gene expression in the GCSF-treated group was similar (Figure 1E). Additionally, the PBS-treated rats on days 3 and 7 post rAION also exhibited similar gene expressions. As stated above, the trend of gene expression is consistent between the PBS- and GCSF-treated groups.

### 2.2. TAFs Involved in Regulation of Cell Death and Proliferation

**Figure 2:**
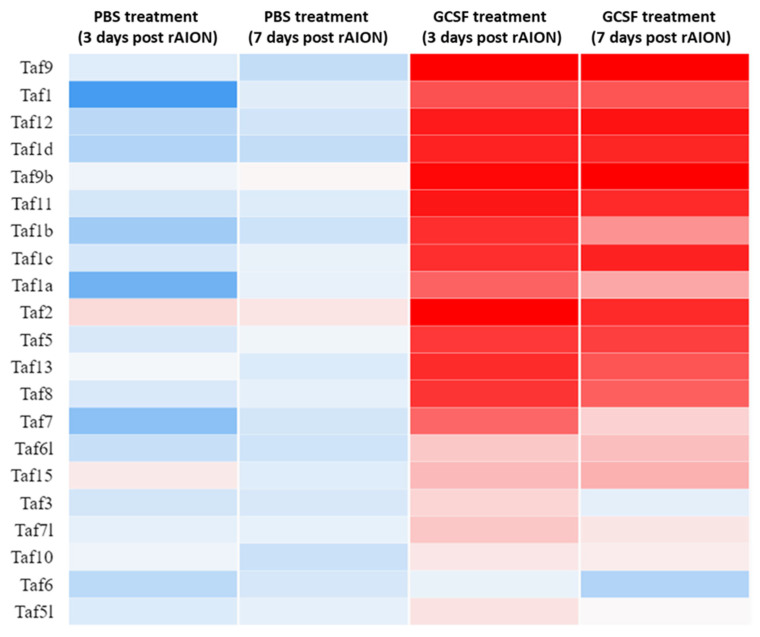
Heatmap of TBP-associated factors expressed in retina samples. After ON infarct, 18 TBP-associated factors were upregulated by GCSF treatment. There were 17 TBP-associated factors that were downregulated by PBS treatment. Genes were clustered into the regulation of cell survival based on gene expression over time. Up- and downregulation are represented in red and blue colors, respectively.

To classify the biological function of differentially expressed genes, we employed a gene ontology (GO) analysis. After GO analysis, many TBP-associated proteins that were classified into the category of regulation of cell death and proliferation. We found that 18 TAFs, including TAF1, TAF1a, TAF1b, TAF1c, TAF1d, TAF2, TAF5, TAF6l, TAF7, TAF7l, TAF8, TAF9, TAF9b, TAF10, TAF11, TAF12, TAF13, and TAF15, were upregulated by GCSF treatment. Additionally, 17 TAFs, including TAF1, TAF1a, TAF1b, TAF1c, TAF1d, TAF3, TAF5, TAF6, TAF6l, TAF7, TAF7l, TAF8, TAF9, TAF10, TAF11, TAF12, and TAF13, were downregulated by PBS treatment (Figure 2). This indicates that the expression levels of many TAFs were suppressed by ON ischemic injury and induced by GCSF treatment.

### 2.3. Network Analysis Revealed That TAFs Directly Interact with TP53 and TBP

**Figure 3:**
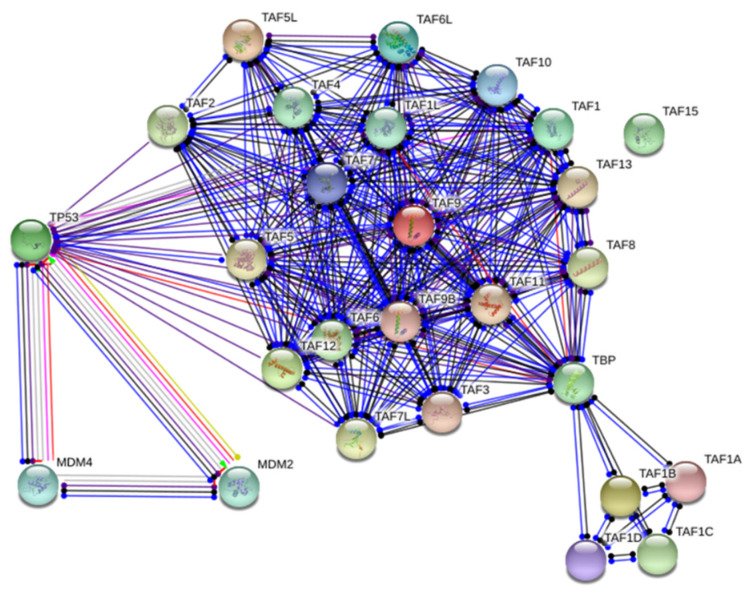
Network analysis of TBP-associated factors. STRING analysis shows that the TBP-associated factors are involved in the known and predicted protein–protein interactions. Network analysis exhibited that many TBP-associated factors directly interact with TP53. The network nodes are proteins. The edges represent the predicted functional associations with 7 differently colored lines representing the existence of the seven types of evidence used in predicting the associations. Red line: the presence of fusion evidence; green line: neighborhood evidence; blue line: cooccurrence evidence; purple line: experimental evidence; yellow line: textmining evidence; light blue line: database evidence; black line: co-expression evidence.

The network analysis summarizes the network of predicted associations for a particular group of proteins. In evidence mode, multiple colored lines indicate the different types of interaction evidence. STRING network analysis exhibited that many TAFs interact directly with TP53, including TAF1, TAF1L, TAF2, TAF3, TAF4, TAF5, TAF6, TAF7, TAF7L, TAF9, TAF9b, TAF10, TAF11, TAF12, and TAF13 (Figure 3). Additionally, TAFs have direct interactions with TBP, including TAF1, TAF1a, TAF1b, TAF1c, TAF1d, TAF1L, TAF2, TAF3, TAF4, TAF5, TAF6, TAF7, TAF7L, TAF9, TAF9b, TAF10, TAF11, TAF12, and TAF13. Among these TAFs, TAF9 was predicted to bind with TP53, and the expression level of TAF9 was dramatically elevated by GCSF treatment and suppressed by PBS treatment. The biological function of TAF9 is involved in gene regulation associated with apoptosis [31]. Thus, we selected TAF9 as a candidate gene to evaluate its function in RGC apoptosis and survival after ON ischemia.

### 2.4. Taf9 Knockdown Impaired the Protective Effect of GCSF on the Visual Function

**Figure 4:**
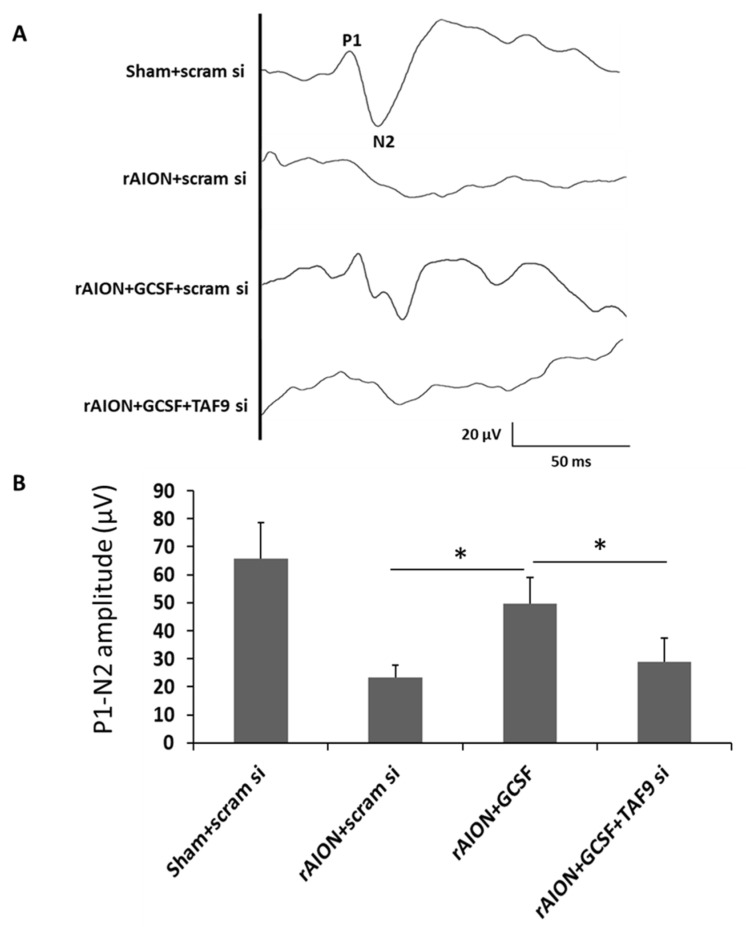
Effect of TAF9 knockdown on FVEP recording in the fourth week after infarct. (**A**) Representative FVEP wavelet in each group. (**B**) GCSF plus TAF9 siRNA treatment reduced the P1-N2 amplitude by 1.71-fold compared to GCSF plus scrambled siRNA treatment in rAION groups (* *p* < 0.05, *n* = 12 per group). Data are expressed as mean ± SD.

To evaluate the role of TAF9 in the protection of visual function in rAION, flash visually evoked potentials (FVEPs) were measured at day 28 post infarct (Figure 4A). On TAF9 knockdown, we found no improvement in visual function despite GCSF treatment. The P1-N2 amplitudes in the sham-operated and scrambled siRNA-treated group (Sham + scram si), rAION induction and scrambled siRNA-treated group (rAION + scram si), rAION induction and GCSF plus scrambled siRNA-treated group (rAION + GCSF + scram si), and rAION induction and GCSF plus TAF9 siRNA-treated group (rAION + GCSF + TAF9 si) were 65.8 ± 12.7 μV, 23.4 μV ± 4.2 μV, 49.5 ± 6.6 μV, and 28.9 ± 8.4 μV, respectively (Figure 4B). Treatment with GCSF plus TAF9 siRNA in the rAION group reduced the P1-N2 amplitude by 1.71-fold compared to that of treatment with GCSF plus scrambled siRNA (Figure 4B, *p* < 0.05). 

### 2.5. Taf9 Knockdown Impaired the Protective Effect of GCSF on RGC Density 

**Figure 5:**
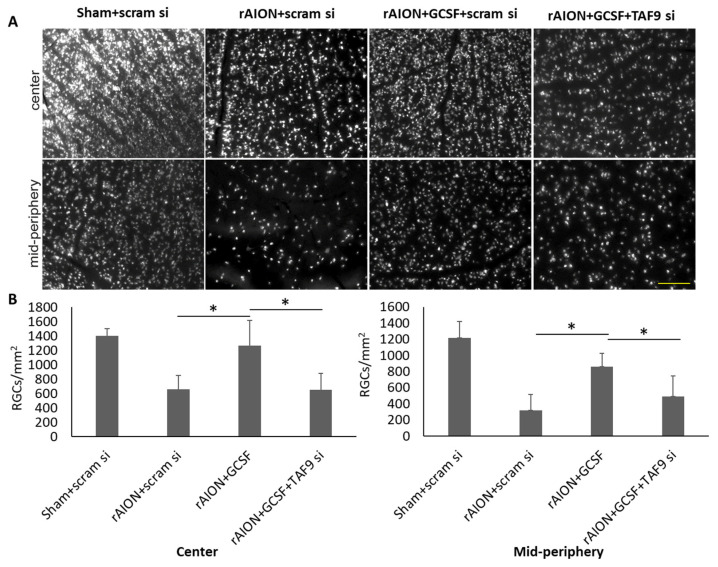
Effect of TAF9 knockdown on RGC density in the fourth week after infarct. (**A**) Representative RGC density of the central and midperipheral retinas in each group. The white spots in the representative figures are the Fluoro-Gold-labeled RGCs in the retina. (**B**) Bar chart showing the RGC density in the rAION induction and GCSF plus TAF9 siRNA-treated rAION group was significantly reduced in the central and midperipheral retinas compared to that in the rAION induction and GCSF plus scrambled siRNA-treated group (* *p* < 0.05, *n* = 12 per group; scale bar = 200 μm).

The survived RGCs were labeled with Fluoro-Gold. In the central retina, the RGC densities in the sham-operated and scrambled siRNA-treated group (Sham + scram si), rAION induction and scrambled siRNA-treated group (rAION + scram si), rAION induction and GCSF plus scrambled siRNA-treated group (rAION + GCSF + scram si), and rAION induction and GCSF plus TAF9 siRNA-treated group (rAION + GCSF+ TAF9 si) were 1402.5 ± 99.1, 655.2 ± 199.6, 1265.1 ± 352.5, and 649.7 ± 227.6 cells/mm^2^, respectively (Figure 5A). In the midperipheral retina, the RGC densities in the sham-operated and scrambled siRNA-treated group (Sham + scram si), rAION induction and scrambled siRNA-treated group (rAION + scram si), rAION induction and GCSF plus scrambled siRNA-treated group (rAION + GCSF + scram si), and rAION induction and GCSF plus TAF9 siRNA-treated rAION group (rAION + GCSF + TAF9 si) were 1219.4 ± 201.3, 319.2 ± 195.8, 863.3 ± 161.3, and 492.9 ± 250.1 cells/mm^2^, respectively (Figure 5). The RGC density in the rAION induction and GCSF plus TAF9 siRNA-treated rAION group was significantly reduced by 1.94- and 1.75-fold in the central and midperipheral retinas, respectively, compared with that in the rAION induction and GCSF plus scrambled siRNA-treated group (Figure 5B, *p* < 0.05). 

### 2.6. TAF9 Knockdown Impaired the Anti-Apoptotic Ability of GCSF

**Figure 6:**
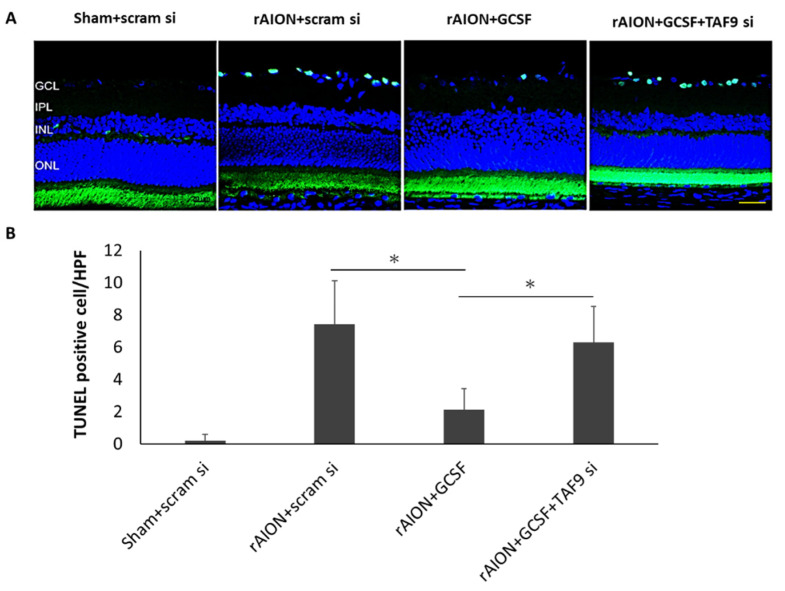
Analysis of RGC apoptosis in the RGC layer through the TUNEL assay in the fourth week after rAION induction. (**A**) Representative images of TUNEL staining. (**B**) Quantification of apoptotic cells per HPF. The number of TUNEL-positive cells in the rAION induction and GCSF plus TAF9 siRNA-treated group significantly increased by threefold compared to that in the rAION induction and GCSF plus scrambled siRNA-treated group (* *p* < 0.05, *n* = 6; scale bar = 50 μm).

The average numbers of TUNEL-positive cells in each high-powered field (HPF, ×400 magnification) in the sham-operated and scrambled siRNA-treated group (Sham + scram si), rAION induction and scrambled siRNA-treated group (rAION + scram si), rAION induction and GCSF plus scrambled siRNA-treated group (rAION + GCSF + scram si), and rAION induction and GCSF plus TAF9 siRNA-treated group (rAION + GCSF + TAF9 si) were 0.2 ± 0.4/HPF, 7.4 ± 2.7/HPF, 2.1 ± 1.3/HPF, and 6.3 ± 2.2/HPF, respectively. The number of TUNEL-positive celsls in the rAION induction and GCSF plus TAF9 siRNA-treated group significantly increased by three-fold compared to that in the rAION induction and GCSF plus scrambled siRNA-treated group (*p* < 0.05), but there was no significant difference between the scrambled siRNA-treated and GCSF plus TAF9 siRNA-treated rAION groups (Figure 6), further suggesting a survival pathway dependent on TAF9. In addition, the number of TUNEL-positive cells in the rAION induction and scrambled siRNA-treated group was significantly higher than that in the rAION induction and GCSF plus scrambled siRNA-treated group (*p* < 0.05). 

### 2.7. TAF9 Knockdown Suppressed GCSF-Induced TP53 and TRIAP1 Expression

**Figure 7:**
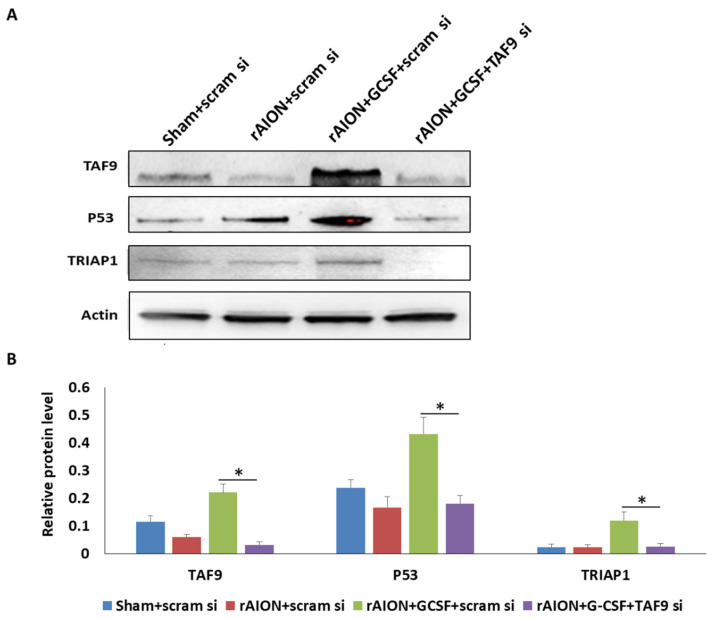
Western blot analysis of TAF9, TP53, and TRIAP1 expression. (**A**) Western blotting image of TAF9, TP53, and TRIAP1. (**B**) Relative protein level of TAF9, TP53, and TRIAP1. Treatment with GCSF plus TAF9 siRNA significantly repressed TAF9, TP53, and TRIAP1 expression by 6.9-, 2.2-, and 4.7-fold compared to treatment with GCSF plus scrambled siRNA in rAION groups (*: *p* < 0.05).

Western blotting confirmed that the rAION induction and GCSF plus scrambled siRNA-treated group exhibited the highest protein level of TAF9 compared with other groups (Figure 7, *p* < 0.05). GCSF plus TAF9 siRNA treatment significantly repressed TAF9 protein expression by 6.9-fold compared to GCSF plus scrambled siRNA treatment (*p* < 0.05). In the rAION induction and GCSF plus TAF9 siRNA-treated group, the TP53 level was reduced by 2.4-fold compared to that in the rAION induction and GCSF plus scrambled siRNA-treated group (*p* < 0.05). One of TP53-regulated genes, the TP53-regulated inhibitor of apoptosis gene 1 (TRIAP1), can inhibit apoptosis through interaction with the APAF1 and heat shock protein 70 (HSP70) complex [32]. Our Western blotting data demonstrate that the TRIAP1 level was reduced by 4.7-fold in the rAION induction and GCSF plus TAF9 siRNA-treated group compared to that in the rAION induction and GCSF plus scrambled siRNA-treated group (*p* < 0.05). In addition, the levels of TAF9, TP53, and TRIAP1 were less in the rAION induction and scrambled siRNA-treated group compared to those in the rAION induction and GCSF plus scrambled siRNA-treated group (*p* < 0.05).

### 2.8. Overexpression of TAF9 Inhibited RGC Death by Modulating TP53–TRIAP1–CASP3 Axis

**Figure 8:**
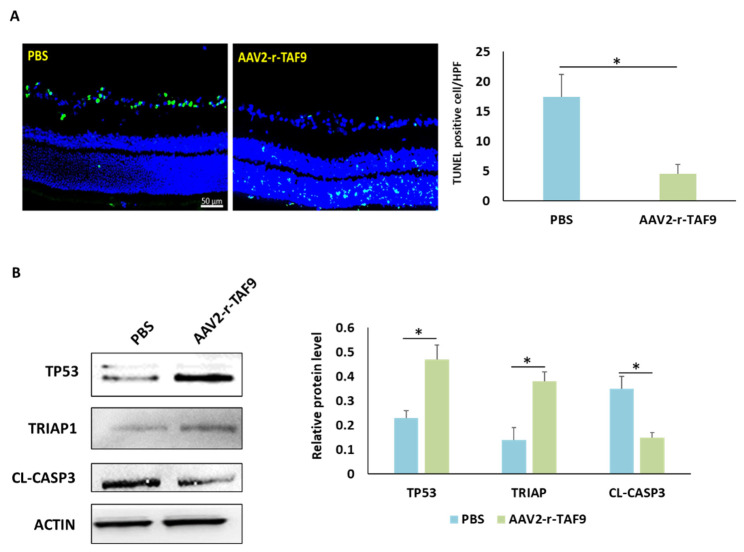
Effects of TAF9 overexpression on RGC apoptosis induced by rAION induction. (**A**) Left: representative images of double-stained apoptotic cells in the ganglion cell layer in each group. The apoptotic cells were stained in green, and the cell nuclei were counterstained with DAPI staining in blue. Right: Analysis of RGC apoptosis between the PBS- and AAV2-r-TAF9-treated groups. (**B**) Western blot analysis of TP53, TRIAP1, and CASP3 expressions in the PBS- and AAV2-r-TAF9-treated groups. (*: *p* < 0.05).

To explore the role of TAF9 in the regulation of RGC death and survival, AAV2-rTAF9 was used to overexpress the TAF9 level in the rAION model. Four weeks after rAION, the numbers of TUNEL-positive cells in the PBS-treated and AAV2-r-TAF9-treated groups were 7.4 ± 2.7/HPF and 2.4 ± 1.7/HPF, respectively (Figure 8A). The number of TUNEL-positive cell was 3.1-fold lower in the AAV2-r-TAF9-treated group than that in the PBS-treated group (*p* < 0.05). Western blotting confirmed that the TP53 and TRIAP1 levels in the AAV2-r-TAF9-treated group were significantly increased, by 2.04- and 2.71-fold, respectively, compared to those in the PBS-treated group (Figure 8B, *p* < 0.05). Moreover, the cleaved-caspase 3 (Cl-casp3) level was reduced by 2.33-fold in the AAV2-rTAF9-treated group compared to that in the PBS-treated group (Figure 8B, *p* < 0.05).

## 3. Discussion

In this study, we conducted a microarray analysis of rat retinas to profile the transcriptomic changes in response to ON ischemic injury and GCSF treatments. Dynamic transcriptome profiling revealed many novel differentially expressed genes involved in the regulation of cell death and proliferation. Among the differentially expressed genes, the TAF protein was shown to be the most regulated and intriguing; this is involved in cell death and proliferation. Furthermore, a subsequent in silico pathway analysis revealed significant interactions between TAFs and TP53. One TP53 coactivator, TAF9, was selected to prove its role in the regulation of RGC death and survival because TAF9 is an apoptosis regulator [33]. TAF9 knockdown effectively reduced the neuroprotective effects of GCSF in the rAION model. We found that TAF9 is a key element in modulating the TP53–TRIAP1–CASP3 pathway. The overexpression of TAF9 inhibited RGC apoptosis in the rAION model. This transcriptomic analysis discovered a novel GCSF-regulated pathway, which is involved in RGC death and survival. 

The differentially expressed genes found in the study are involved in the regulation of RGC death. Notably, ON ischemia influenced 702 and 179 transcripts on days 3 and 7 post rAION, respectively. We found that the numbers of ON ischemia-influenced genes gradually reduced from days 3 to 7 post rAION. These data indicate that a dramatic change in transcription occurs in the acute stage, but this transcriptional change returns to normal in the subacute stage. A similar observation was found in our previous study, whereby vascular permeability was highly increased in the acute stage and reduced in the subacute stage after ON infarct [7]. Taken together, we consider that ON ischemia may cause severe pathological changes in the acute stage, and the natural course of recovery may be started in the subacute stage. Therefore, the therapeutic window should be focused on the acute stage in ON ischemia. As expected, our previous findings also demonstrated that early treatment with GCSF or methylprednisolone provided good neuroprotective effects in the rAION model [7,34]. 

Comparing the GCSF-treated groups with the PBS-treated groups, GCSF treatment constantly influenced >3000 transcripts for 7 days, but the PBS-treated rats showed gradually reduced transcriptional changes from the acute to subacute stage. This indicates that immediate treatment with GCSF can influence several genes to trigger the rescue actions after ON ischemia. These remarkable transcriptional changes provide informative messages in discovering the key pathways involved in RGC survival. In this transcriptomic analysis, we found that many TBP-associated proteins were suppressed by ischemic insult, but induced by GCSF treatment. These TAFs were involved in the regulation of RGC death and survival. At the molecular level, gene expression is regulated by many core transcriptional complexes, such as TFIID, along with different cofactors [35]. Previous studies revealed TFIID as an integral component of the core transcriptional machinery for RNA polymerase II in mRNA-encoding genes [35,36], and demonstrated that it is assembled with TBP and multiple TAFs [37]. To date, many TAFs and several tissue-specific variants have been characterized [38]. Some genetic studies have revealed the complex role of TFIID in controlling tissue-specific and context-dependent transcriptional processes, proving the existence of different TFIID complexes and tissue-specific TAFs [39,40,41,42,43]. TFIID subunits regulate many cellular processes in tissue-specific manners, which facilitates research into TAF involvement in moderating biological functions, including proliferation, differentiation, apoptosis, metastasis, and hormone response [44].

After the network analysis, we found that TAF9 was predicted to interact with TBP and TP53. In addition, TAF9 was highly upregulated by GCSF treatment at days 3 and 7 post rAION. This implies that TAF9 plays an important role in modulating RGC death and survival via the P53 signaling pathway. TAF9 was reported to be a crucial P53 coactivator for the stabilization and activation of P53 [28,30]. TAF9 inhibits the MDM2-mediated degradation of p53 by reducing MDM2 binding to p53 [30]. A previous study also demonstrated that one TFIID complex lacking TAF9 in Hela cells causes apoptosis [45]. The interruption of interactions between Hedgehog transcription factors (Gli proteins) and TAF9 reduces Gli/TAF9-dependent transcription, suppresses cancer cell proliferation, and reduces xenograft growth [33]. As mentioned above, we hypothesize that the high TAF9 level activates the P53 pathway to inhibit RGC apoptosis after ON infarct. To verify our hypothesis, the TAF9-knockdown experiment was performed to discover the role of TAF9 in the regulation of RGC death and survival. As expected, TAF9 knockdown effectively reduced the protective effects of GCSF in the rAION model. Therefore, we have demonstrated that TAF9 plays a key role in RGC protection after ON ischemia. 

Cell apoptosis is manipulated on multiple levels by the sequence-specific transcription factor TP53, with >100 genes existing TP53 binding sites [46]. Moreover, the function of these genes remains unclear. One of these genes is the TP53-regulated inhibitor of apoptosis gene 1 (TRIAP1), which has a p53 binding site within its coding sequence and is upregulated in many cancer cells [47]. TRIAP1 was reported to protect cancer cells from apoptosis through interaction with HSP70 or the repression of cyclin-dependent kinase inhibitor 1 (p21) [48,49]. Recent findings have demonstrated that TRIAP1 contributes to the resistance of apoptosis in a mitochondria-dependent manner [50,51]. Based on this evidence, we further evaluated the relationship among TAF9, P53, TRIAP1, and CASP3 in the rAION model. Remarkably, immunoblotting data have demonstrated that TAF9 overexpression prevented TP53 degradation and increased TRIAP1 expression in the rAION model. Additionally, we found that TAF9 overexpression reduced apoptotic RGCs and caspase 3 level after ON infarct. Taken together, we can suggest that TAF9 plays a key role in the protection of ischemia-induced RGC apoptosis by modulation of the TP53–TRIAP1–CASPS3 axis.

**Figure 9:**
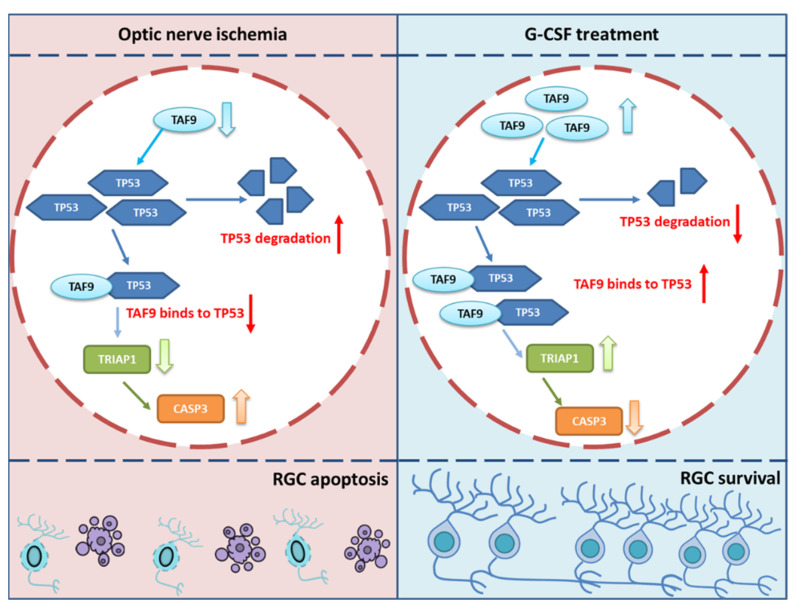
The graphic abstract of the protective mechanism of G-CSF after ON infarct. G-CSF treatment induces the level of TAF9 to prevent TP53 degradation. The binding complex of TP53 and TAF9 induces the level of TRIAP1 to inhibit the level of cleaved CASP3; as a result, the RGC apoptosis is inhibited by treating G-CSF. (↑: increase; ↓: decrease).

Therefore, our transcriptomic analysis found a novel signaling pathway to elucidate the anti-apoptotic effects of GCSF on RGCs. In this novel signaling pathway, TAF9 is a key element in the modulation of the TP53–TRIAP1–CASPS3 axis for preventing RGC death (Figure 9). All the evidence suggests that TAF9 is a potential target in developing a new drug for NAION treatment.

## 4. Materials and Methods

### 4.1. Study Design

In examining the transcriptome profiles in the retina, the rAION-inducted rats were treated by PBS or GCSF. The GCSF-treated group and the PBS-treated group received a subcutaneous injection of G-CSF (100 μg/kg body weight/day in 0.2 mL of saline; Takasaki Pharmaceutical Plant, Tokyo, Japan) or PBS (0.2 mL) once daily for 3 days, respectively. At day 3 post rAION, the retina samples were collected in the PBS-treated group (*n* = 3) and GCSF-treated group (*n* = 3). At day 7 post rAION, the retina samples were again collected in the PBS-treated group (*n* = 3) and GCSF-treated group (*n* = 3). The retina samples in the sham-operated group (*n* = 3) were also collected. All retina samples were used to extract the mRNA. The mRNA samples were analyzed by RNA microarray to profile the transcriptome in each group. The differentially expressed genes were classified by GO analysis. Among the differentially expressed genes, the TBP-associated proteins were classified into the function of cell growth. Therefore, the TBP-associated proteins were selected to predict the protein-to-protein interaction by network analysis (STRING 9.0). The TP53 was predicted to interact with many TBP-associated proteins in the network analysis. One of the TBP-associated proteins, TAF9, was selected to be the target protein to verify the function in the regulation of cell death and survival. TAF9 knockdown and overexpression experiments were performed in the rAION model. The rAION-inducted rats were intravitreally treated with scrambled siRNA (50 pmol; GeneDirex, Keelung, Taiwan), GCSF plus scrambled siRNA (50 pmol; GeneDirex), and GCSF plus TAF9 siRNA (50 pmol; GeneDirex) to evaluate the visual function (*n* = 12 in each group), RGC density (*n* = 12 in each group), and apoptotic RGCs (*n* = 6 in each group). The TAF9, TP53, and TRIAP1 levels were evaluated in each group (*n* = 6) using Western blot analysis. Moreover, the AAV2-mediated overexpression of TAF9 was intravitreally administered before rAION induction to examine the anti-apoptotic ability of TAF9 in the rAION model. The number of apoptotic RGCs was evaluated in the AAV2-r-TAF9-treated group (*n* = 6) and PBS-treated group (*n* = 6). The TP53, TRIAP1, and cleaved-CASP3 levels were determined in the AAV2-r-TAF9-treated group (*n* = 3) and PBS-treated group (*n* = 3).

### 4.2. Animals

Male Wistar rats were used in the study. The rats were aged 6–8 weeks with body weights of 150–180 g. Animal care and experimental procedures were performed in accordance with the Association for Research in Vision and Ophthalmology Statement for the Use of Animals in Ophthalmic and Vision Research, and the Institutional Animal Care and Use Committee (IACUC) at the Tzu Chi Medical Center approved all animal experiments. 

### 4.3. rAION Induction

The procedure of rAION induction was described in our previous study [52]. Before general anesthesia, all rats were administered Mydrin-P (Santan, Osaka, Japan) and Alcaine (Alcon, Fort Worth, TX, USA) eye drops for pupil dilation and topical anesthesia, respectively. Subsequently, the rats were injected intramuscularly with a mixture of ketamine (40 mg/kg body weight, Pfizer, Tadworth, UK) and xylazine (4 mg/kg body weight; Sigma, St. Louis, MO, USA) for general anesthesia. For photosensitization, 2.5 mM Rose Bengal diluted in PBS (1 mL/kg of body weight) was administered intravenously before laser application. After rose bengal injection, the optic disc was immediately exposed to an argon green laser system (MC-500 Multi-color laser, Nidek Co., Ltd., Tokyo, Japan, setting: 532 nm wavelength, 500 μm size and 80 mW power) for 12 1-second pulses. A laser fundus lens (Ocular Instruments, Inc., Bellevue, DC, USA) was used to target the optic disc. At the end of experiment, TobraDex eye ointment (Alcon-Couvreur, Puurs-Sint-Amands, Belgium) was applied to the eyes of all experimented rats.

### 4.4. RNA Microarray Analysis (Quality Check, Annotation, and Ontology)

The retina samples were collected at day 3 and day 7 post rAION. Total RNA was isolated from retina homogenate using TRIzol reagent (Invitrogen) according to the manufacturer’s instructions. Complementary DNAs were synthesized using reverse transcriptase kit. RNA microarray analysis was performed using the Rat OneArray kit according to the manufacturer’s protocol. Clustering and principal component analysis were performed to determine the differences among biological sample replicates and their treatment conditions. Raw intensities were normalized with the median scaling normalizing method, and covariance was determined by the error model of the Rosetta Resolver system. Normalized intensity was transformed to the log2 ratio (fold change). Gene annotation was performed with reference to NCBI RefSeq Release 57. EnsEMBL released 70 cDNA sequences and rattus_norvegicus_core_70_5b annotations. Differentially expressed genes that showed both a log2 ratio (fold change) >1 and *p* < 0.05 were considered candidate genes. 

### 4.5. Flash Visual-Evoked Potentials (FVEPs)

FVEP measurements were performed as described in our previous study [52]. After general anesthesia, the sagittal region of the skull was opened in the rats. The 4 mm screw implants were passed through the skull to approximately 1.5 mm and placed at the frontal cortex and primary visual cortex regions of both hemispheres using stereotaxic coordinates. A visual electrodiagnostic system (Diagnosys LLC, Lowell, MA, USA) was used to measure the FVEP. The number of average sweeps was 64 for each rat. A comparison of the average amplitude of the P1-N2 wave in each group was made to evaluate visual function.

### 4.6. Retrograde Labeling of RGCs and Measurement of RGC Density

RGCs were labeled as described in our previous study [52]. Briefly, retrograde tracer dye fluorogold was injected into the superior colliculus one week before the rats were euthanized. One week after labeling, the rats were euthanized, and retinas were carefully flat-mounted. The central and midperipheral regions in the retina were examined under a 200× fluorescence microscope (Axioplan 2 imaging, Carl Zeiss, New York, NY, USA) with a built-in filter set (excitation filter, 350–400 nm; barrier filter, 515 nm) and connected digital imaging system. Six randomly selected areas in the central and midperipheral regions were used to calculate the number of RGCs in the central and midperipheral regions of each retina. The number of RGCs was calculated using the ImageMaster 2D Platinum Software V 7.0 (GE Healthcare, Chicago, IL, USA).

### 4.7. Retinal Tissue Preparation and Sections

After euthanizing, the rat eyeballs were fixed in 4% paraformaldehyde overnight. The eyecups and ONs were separated and transferred to 30% sucrose solution; the samples were incubated at 4 °C until they settled at the bottom of the tubes. The retina cross-section and ON longitudinal sections of 10 μm were obtained by using a cryostat-microtome.

### 4.8. TUNEL Assay

To determine the apoptotic RGCs in the retinal section, the TUNEL assay kit (Click-iT™ Plus TUNEL Assay, Invitrogen, Waltham, MA, USA) was used to stain the apoptotic cells in the ganglion cell layer. Nuclei were stained with 4t,6-diamidino-2-phenylindole (DAPI). The TUNEL-positive cells in the ganglion cell layer of each section were counted in 10 HPF (400×), and an average of six sections per group was used for further statistical analysis.

### 4.9. Western Blotting Analysis 

After euthanizing, the rats’ eyes were enucleated. The retinas were homogenized in lysis buffer. The protein sample was separated on 10% bis-acrylamide gel. The proteins were transferred to polyvinylidene difluoride (PVDF) membranes. The membranes were blocked with 5% non-fat dry milk for 1 h. The membranes were incubated with Taf9, TP53, TRIAP1, CASP3 and ACTIN antibody at 4 °C for 12–16 h, followed by incubation with a secondary antibody conjugated to HRP against the appropriate host species for 1 h at room temperature. Then, the membranes were developed using enhanced chemiluminescent (ECL) substrate. Membranes were exposed to a Western blot analyzer, and the relative density was calculated using image master platinum software V 7.0 (GE Healthcare, Chicago, IL, USA). 

### 4.10. Intravitreal Injection of AAV2-rTAF9

The AAV vectors AAV2-CMV-rTAF9 (NovoPro Biotechnology, Shanghai, China) used in this study consisted of the AAV2 capsid, the CMV promoter, and codon-optimized rat TAF9 cDNA. Briefly, rats were anesthetized with ketamine/xylazine and the injected eye was numbed with Alcaine drops. After the general anesthesia, we selected an area on the sclera, devoid of blood vessels, and slowly injected 3 μL of AAV vector preparation into the vitreous cavity for 30 s using a 10 μL Hamilton syringe with a 30 G needle. Tobradex eye ointment was applied after injection to prevent infection in each rat.

### 4.11. Statistical Analysis

All statistical analyses were performed using IBM SPSS software. The data are presented as mean ± standard deviation. A Mann–Whitney U test was used for comparisons between groups. *p*-values < 0.05 were considered statistically significant, with * representing *p* < 0.05.

## 5. Conclusions

In conclusion, we proved that GCSF has an anti-apoptotic effect on RGCs after ON infarct via modulating the TAF9–TP53–TRIAP1–CASPS3 axis. TAF9 is highly induced by GCSF to prevent MDM2-mediated degradation of TP53. The binding complex of TAF9 and TP53 upregulates the level of TRIAP1 to inhibit RGC apoptosis after ON infarct (Figure 9). However, GCSF treatment may result in leukocytosis, fever, muscle pain, and joint pain in patients with NAION. Induction of TAF9 and TRIAP1 in the retinal cells using the transfection system may provide therapeutic effects similar to GCSF, and may preclude the side effects from GCSF treatment. Thus, we believe that TAF9 and TRIAP1 may be the potential targets of gene therapy for NAION patients in the future. 

## Data Availability

Data as reported in this study are available from corresponding author upon reasonable request.
